# Study of the corrective forces applied by a dynamic derotation brace (DDB)

**DOI:** 10.1186/1748-7161-7-S1-O31

**Published:** 2012-01-27

**Authors:** I Loukos, C Nicolopoulos, C Zachariou

**Affiliations:** 1 Medical School, National & Capodistrian University of Athens Anoixi, Greece; 2 ORTHO-FOOT Center, Athens, Greece; 3 KAT Hospital of Athens, Greece

## Background and purpose

The direct forces exerted by the brace pad were analysed, on 44 idiopathic scoliotic patients (38 girls, 6 boys) treated with a DDB [1]. Twenty seven patients had a single right thoracic curve and 17 had a single left or right thoracolumbar curve. The aim was the analysis of the corrective forces, exerted at the skin-brace interface, by altering the posture, activity and strap tension [2-7].

## Materials and methods

We used the F-Socket 9801 pressure sensor and the MatScan Research BETA STAM v. 6.30 software (TekScan, Boston MA, USA), and measurements were taken in nine body postures. The patients were divided into three groups: those who were wearing for first time the brace, those who changed their brace with a new one and those who made corrections on their brace.

## Results

Changes in strap tension, body posture, resulted in statistically significant alterations of the interface pressure on the pads and thus of the resultant forces exerted on the patient’s body by the pads. Comparing the three groups in relation with the magnitude of the mean exerted force, the correction of the brace caused the highest mean exerted force.

## Conclusions

Even though the TekScan system does not provide direct information on the correction of spinal curvature, it appears to be a useful tool in the treatment of scoliotic patients. Also, the analysis of the corrective forces seems to be very helpful trying to achieve brace’s optimal fit and the same time the best therapeutic result for the patient.
